# Sheep models for evaluation of novel patch and prosthesis material in vascular surgery: tips and tricks to avoid possible pitfalls

**DOI:** 10.1186/s13028-018-0397-1

**Published:** 2018-07-05

**Authors:** Karina Schleimer, Houman Jalaie, Mamdouh Afify, Anna Woitok, Mohammad Esmaeil Barbati, Konrad Hoeft, Michael Jacobs, Rene H. Tolba, Julia Steitz

**Affiliations:** 1 Clinic of Vascular and Endovascular Surgery, Faculty of Medicine, RWTH Aachen University, 52074 Aachen, Germany; 2 Institute for Laboratory Animal Science, Faculty of Medicine, RWTH Aachen University, Pauwelsstrasse 30, 52074 Aachen, Germany; 3 Department of Pathology, Faculty of Veterinary Medicine, Cairo University, Cairo, 12211 Egypt

**Keywords:** Arteriovenous prosthetic shunt, Carotid artery, External jugular vein, Graft-interposition, Patch-angioplasty, Sheep models, Vascular surgery

## Abstract

**Background:**

In vascular surgery, novel synthetic prosthesis materials for patch-angioplasties, interpositions, bypasses and shunts are continuously under development and optimization. The characteristics of an ideal vascular prosthesis would display long-term patency, biocompatibility, durability, low porosity, lack of stich hole bleeding, ease of handling, kink resistance, infection resistance and reasonable costs. The aim of this study was to establish and report a reliable sheep model including potential pitfalls where those parameters could be analyzed. Before surgery, sheep were acclimatized for 4–8 weeks, during which parasite infections were treated and blood and serum parameters monitored. Twenty-four sheep underwent surgery, and carotid patch-angioplasties (n = 12), graft interpositions (n = 6) or arteriovenous prosthetic shunts (n = 6) were implanted. Half of the animals in each group were sacrificed after 2 weeks and the other half after 8 weeks. The implants were analyzed for patency, endothelialization, thrombogenicity and biocompatibility by clinical observation, blood flow measurement and pathological and histopathological (H&E, EvG) as well as immunohistochemical (Ki67, CD31) evaluations.

**Results:**

Health monitoring of the sheep revealed a parasitic burden with endoparasites in all animals. Some animals showed thereby infestations in the bile duct causing fibrotic cholangitis with calcifications in the liver. In addition, sarcosporidia were detected in histopathological specimen of the heart in all animals. Parasitic burden correlated with blood counts and serum bilirubin levels. Both were significantly reduced by albendazole treatment within the acclimatization time. Patches, interposition grafts, and straight shunts were successfully implanted bilaterally in all animals. The total average operation time was 136 ± 21 min. Most animals (23/24) showed good patency rates and general condition after implantation. Pathological and histopathological/immunohistochemical analyses were suitable to determine thrombogenicity, endothelialization, cellular/fibroblastic proliferation, biocompatibility, inflammatory cell infiltration, and thickness of neointima in the prosthesis material.

**Conclusions:**

We have developed a suitable experimental protocol with standardized and successful anesthesia- and surgical-procedures for patch-angioplasty, graft interposition, and arteriovenous prosthetic shunts. This sheep model allows testing of new prosthetic materials for biocompatibility, thrombogenicity, and endothelialization.

## Background

In vascular surgery, synthetic prosthesis material is globally used for patch-angioplasties, interpositions, bypasses and straight shunts. Such prosthesis material is required either when suitable autologous veins are not available or in order to save time during critical operations. Additionally, the current standard procedure requires prosthesis straight or loop shunts, if there is not enough time for the maturation of a native shunt. An ideal vascular prosthesis would display the following characteristics: long-term patency, biocompatibility, durability, low porosity, lack of stich hole bleeding, ease of handling, kink resistance, and resistance towards infection; ideally all at a reasonable cost. In 1957, DeBakey introduced the first polyethylene terephthalate (PET; e.g. Dacron) prosthesis in aortic surgery [1]. In 1976, the first successful clinical implantation of polytetrafluoroethylene (PTFE) grafts was reported [2]. Currently, Dacron and PTFE are the most widely used prosthesis materials. Compared to veins, however, they have a higher risk of anastomotic neointimal hyperplasia, thrombosis, and infection. In spite of significant efforts by industrial and academic research groups over the past 60 years, an ideal synthetic vascular graft has not been developed. Hence, the development of an optimal vascular prosthesis is a challenging issue in vascular surgery research [3]. Evaluation of new vascular prosthesis material in preclinical animal studies is required to assess biocompatibility, thrombogenicity, endothelialization as well as the capacity of the prosthesis to maintain a physiologic function in the circulatory system. It is also a prerequisite for registration of a medical device, as demanded by the Food and Drug Administration (FDA) and European Medical Devices Directive (MDD).

In the context of such animal experiments, the following questions need to be clarified:

- Which animal species is suitable?
- In which anatomical region should the operation be performed and what are the anatomical features of this animal species?
- How do the animals have to be pretreated?
- Which anesthesia and surgical protocols should be used?
- Which analyses or methods are adequate to address functionality, biocompatibility, thrombogenicity, and endothelialization of the tested prosthesis material?
- Which time points are ideal for follow-up evaluations?

To develop an ideal vascular prosthesis, many studies have been carried out in different species (specifically in sheep and pigs), each with different surgical procedures, examination methods, and observation periods [4–29].

We have developed a suitable experimental protocol, taking into account the available studies as well as our own expertise in vascular surgery, experimental animal medicine and animal pathology. The aim of this article is to present our standardized and successful anesthesia- and surgical-procedures for patch-angioplasty, graft interposition, and arteriovenous prosthetic shunts in carotid arteries and jugular veins of sheep. We describe in detail the pre- and post-treatment procedures as well as the investigations carried out on the animals. We give tips and tricks in order to avoid possible pitfalls as well as unnecessary loss of experimental animals and to improve animal welfare.

## Methods

### Study design

A total number of 24 sheep underwent surgery, as shown in Table 1: Carotid patch-angioplasties were performed in 12 sheep, graft interpositions in 6 sheep and arteriovenous prosthetic shunts between the common carotid artery and the external jugular vein in 6 sheep. We implanted the grafts bilaterally in the common carotid arteries, with a total of 48 surgeries performed. In each treatment group, half of the animals (6, 3 and 3 respectively) were euthanized at 2 weeks and the other half at 8 weeks after implantation.

**Table 1:** Study design, treatment groups and dropout rate

Treatment group	Animal number	Number of prostheses	Procedure duration (weeks)	Drop out
Patch	6	12	2	0/6
	6	12	8	0/6
Graft interposition	3	6	2	1/3 (thrombo-embolic complication)
	3	6	8	0/3
Shunt	3	6	2	0/3
	3	6	8	0/3

**Fig. 1:**
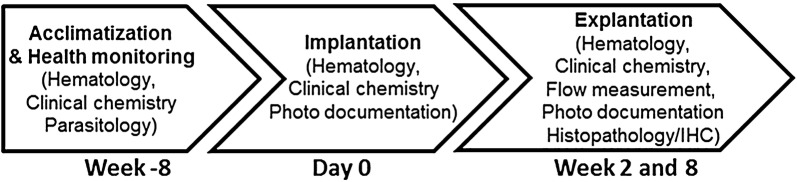
Time axis of experimental procedures and analyses

Figure 1 depicts the time axis of the experimental procedures and analyses: Initially, a clinical examination and hematology, clinical chemistry and parasitology were performed to determine animal health status. After 4–8 weeks of acclimatization, prostheses (patch, interposition graft or shunt) were implanted. Animals were clinically examined weekly for patency of the carotid artery and general health. 2 or 8 weeks after implantation, the animals were euthanized and prosthesis material was explanted. At this time, the following parameters were examined: clinical health, hematology and clinical chemistry parameters, blood flow in the common carotid arteries, gross and microscopic lesions and immunohistochemistry.

### Animals

Twenty-four apparently healthy female Swifter sheep were obtained from a hygienic controlled breeding facility (Zootechnisch Centrum, 3360 Lovenjoel, Belgium). The sheeps’ average body weight (BW) was 59 ± 6 kg (mean ± standard deviation of mean (SD)). The animal protocol was approved by the Governmental Animal Care and Use Committee (LANUV, North Rhine Westphalia, Recklinghausen, Germany, AZ 84-02.04.2012.A023). All animals were housed, cared for and operated in accordance with the German legislation governing of animal studies following the Guide for the Care and Use of Laboratory Animals (NIH publication, 8th edition, 2011) and the 2010/63/EU Directive on the protection of animals used for scientific purposes (Official Journal of the European Union, 2010).

### Health monitoring and acclimatization

After a first clinical check by a veterinarian, EDTA blood for hematology and blood for the collection of serum were taken. Serum samples were obtained by centrifugation of blood samples at room temperature with 2500×*g* for 10 min and stored at − 20 °C for later analysis. Blood cell counts were performed using the Celltac α MEK-6450 K (Nihon Kohden Europe) and microscopy of blood smears after Wright eosin methylene blue staining for differential hemogram. Serum parameters (ALB, ALP, ALT, AMYL, AST, BUN, Ca, CHOL, CK, Cl, CREA, CRP, GGT, GLU, K, LAC, LDH, LIP, Mg, Na, PHOS, TBIL, TP, TRIG, UAC) were analyzed with the Vitros 250/350 (Ortho-Clinical Diagnostics, Neckargmünd, Germany) clinical chemistry automated system. Native fecal and floatation samples were investigated via light microscopy for detection of endoparasites. A single dose of 7.6 mg/kg albendazole (Valbazen^®^ 1.9%, Lilly Deutschland GmbH, Bad Homburg, Germany) was administered orally to all sheep. All sheep were acclimatized at least 4 weeks prior to experiments. The animals were housed in a certified facility with humidity- and temperature-controlled environment and a 12:12 h light:dark cycle. Animals were fed hay, regular diet pellets (0.3–0.4 kg/animal/day) (SSniff Spezialdiäten GmbH, Soest, Germany) and water ad libitum.

### Anesthesia

All sheep were fasted for 12 h with access to water ad libitum until animals were pre-medicated i.m. with a pre-mixed solution of 0.16 mg/kg atropine (ATROPINSULFAT 100 mg, Dr. Franz Köhler Chemie GmbH, Bensheim, Germany) and 0.2 mg/kg xylazine (XYLAZIN 2%, Ceva Tiergesundheit GmbH, Düsseldorf, Germany). An 18–20 G intravenous catheter was inserted into the auricle vein and a prophylactic dose of antibiotic cefuroxim (1.5 g i.v., Cefuroxim, Fresenius Kabi Deutschland GmbH, Bad Homburg, Germany) was administered. General anesthesia was induced i.v. with 2.6 mg/kg propofol (Propofol 1% MCT Fresenius, Fresenius Kabi Deutschland GmbH, Bad Homburg, Germany). Thereafter, the sheep were intubated with an 8.5 Ch endotracheal tube, positioned in a supine position and the endotracheal tube was connected to an inhalation device (Cato, Fa. Dräger, 23558 Lübeck, Germany). General anesthesia was maintained with 1.5 vol% isoflurane inhalation and intravenous infusion of 0.02 mg/kg/h fentanyl (FENTANYL, ROTEXMEDICA GmbH, Trittau, Germany). During anesthesia, sheep were ventilated with a tidal volume of 7–9 mL/kg BW (f: 16/min, PEEP: 5 mmHg). The sheep received a urinary catheter and a stomach tube and the vital functions were monitored. The average duration of surgery was 136 ± 21 min, during which a continuous infusion of Ringer’s solution (Ringer Lösung, B. Braun Melsungen AG, Melsungen, Germany), with 10 mL/kg/h was administered.

### Implantation

**Fig. 2:**
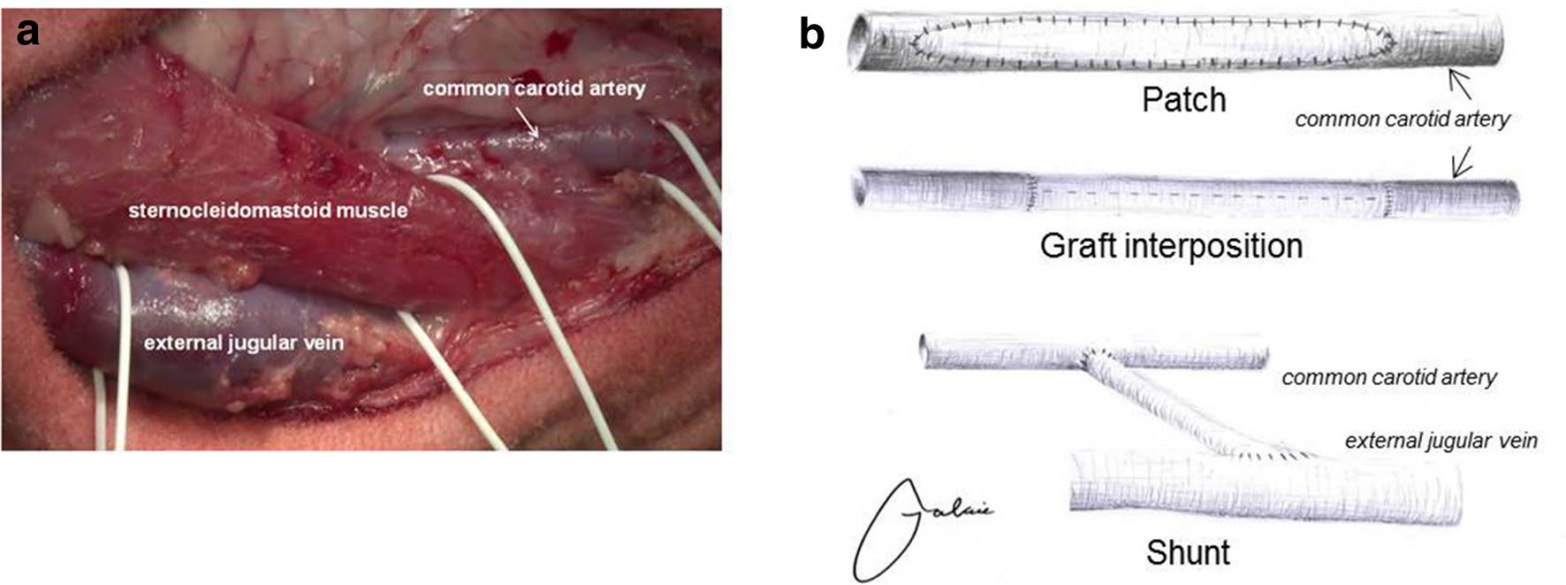
**a** Cervical anatomy of the sheep. **b** Schematic depiction of carotid patch angioplasty, graft interposition and straight shunt between the common carotid artery and the external jugular vein

The neck was prepared for aseptic surgery and a 15 cm longitudinal skin incision was made parallel to the medial border of the sternocleidomastoid muscle. The subcutaneous tissue and the platysma were divided and the medial border of the sternocleidomastoid muscle was mobilized and retracted laterally with self-retaining retractors. The common carotid artery was carefully exposed and the vagus nerve was identified and preserved and an 8 cm segment of the common carotid artery was dissected. Figure 2a depicts the cervical anatomy of the sheep with sternocleidomastoid muscle, common carotid artery and external jugular vein. Adapted from the surgery we usually perform in humans, we carried out the following operations in sheep:

#### Patch angioplasty

**Fig. 3:**
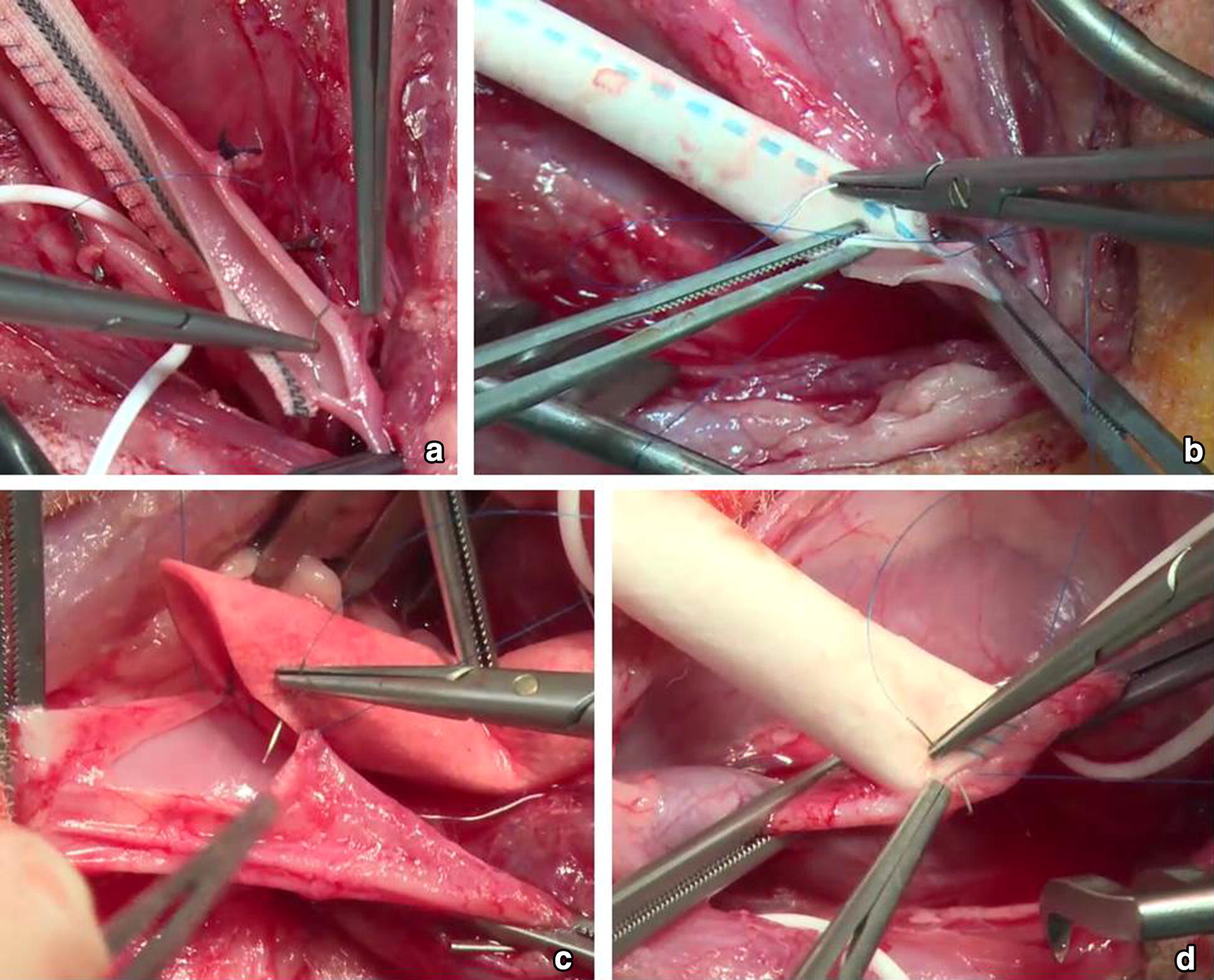
**a** Patch angioplasty of the common carotid artery. **b** End-to-end anastomosis between the prosthesis and the common carotid artery during graft interposition. **c** End-to-side anastomosis between the prosthesis and the external jugular vein during creation of a straight shunt. **d** End-to-side anastomosis between the prosthesis and the common carotid artery during creation of a straight shunt

After intravenous administration of heparin (5000 IU, Heparin-Natrium, B. Braun Melsungen AG, Melsungen, Germany), the common carotid artery was clamped cranially and caudally with peripheral vascular clamps. A 6 cm longitudinal arteriotomy was performed, the lumen of the carotid artery was rinsed with heparinized saline and the artery was closed by patch angioplasty. The fusiform patch (Dacron Fluoropassiv, Gelatin impregnated thin wall, knitted Carotid Patch, 920875FT, 6 mm width × 6 cm length), was soaked in heparinized saline solution (10,000 IU/200 mL) for at least 5 min prior to implantation. It was sewn in accurately with a 6.0 polypropylene (Prolene 6-0 TF1, Johnson & Johnson Medical GmbH, Norderstedt, Germany) running suture (Figs. 2b, 3a). We started the suture line at the superior end of the arteriotomy in the carotid artery. When the suture line was nearly completed, the vascular clamps were briefly released to flush air or debris out of the artery. The carotid artery was rinsed with heparinized saline, before the arteriotomy was finally closed and the blood flow was restored.

#### Graft interposition

After intravenous administration of heparin (5000 IU), the common carotid artery was clamped cranially and caudally with vascular clamps. An excision of a segment of about 6 cm length of the common carotid artery was followed by an interposition of the vascular prosthesis (Gore-Tex Vascular Graft, VT 06070L, 6 mm inner diameter, 6 cm length): After rinsing the arterial stump, the oblique end-to-end anastomosis between the prosthesis and the cranial arterial stump was performed with a continuous running suture in a parachute-technique (6-0 Prolene TF1, Johnson & Johnson Medical GmbH). Subsequently, the cranial clamp was removed and the anastomosis released. The graft was flushed and rinsed with heparinized saline before it was clamped with a Fogarty clamp. Now, the oblique end-to-end anastomosis between the prosthesis and the caudal stump of the common carotid artery was performed in the same manner (Figs. 2b, 3b). As soon as the anastomosis was nearly completed, the vascular clamps were briefly released to flush air or debris out of the artery. The carotid artery and the graft were rinsed with heparinized saline, before the anastomosis was completed and released.

#### Arteriovenous prosthetic shunt

In contrast to humans, in whom the internal jugular vein is the major cervical vein, the external jugular vein is the dominant cervical vein in sheep (Figs. 2b, 3c/d). Therefore, an arteriovenous prosthetic shunt is performed between the common carotid artery and the external jugular vein. Additionally to the preparation of the common carotid artery, we exposed the external jugular vein at the posterior border of the sternocleidomastoid muscle. After i.v. administration of heparin (5000 IU), the caudal part of the external jugular vein was clamped with two vascular clamps and incised for 15 mm. Next, the end-to-side anastomosis between the external jugular vein and the graft (Gore-Tex Vascular Graft, VT 06070L, inner diameter: 6 mm, length: approximately 6 cm) was performed in a running suture technique using a polypropylene 6–0 vascular suture (6–0 Prolene TF1, Johnson & Johnson Medical GmbH, Fig. 3c). The clamps were released and the graft was flushed and rinsed with a heparin saline solution. Subsequently, a Fogarty clamp was placed onto the graft beneath the anastomosis. Afterwards, the graft was passed through a tunnel under the sternocleidomastoid muscle and anastomosed end-to-side with the cranial part of the common carotid artery, which had been clamped with two vascular clamps and incised for 6 mm just previous to the anastomosis (Fig. 3d). Before completion of the anastomosis, all clamps were released and the anastomosis was flushed and rinsed with heparin saline solution. Then, the anastomosis was completed and released by removal of the clamps. Kinking of the grafts was avoided by implanting them under mild tension.

In all animals, a second dose of heparin (2500 IU) was administered i.v. before the carotid artery of the opposite site was clamped and operated in the same manner.

At the end of surgery, the wound was closed in two layers after careful hemostasis. The platysma muscle was sewn with 2.0 Vicryl (Johnson & Johnson Medical GmbH). The skin incision was closed with a 2–0 polyamid thread (2–0 Ethilon, Johnson & Johnson Medical GmbH).

### Postoperative care

After the sheep had recovered their swallowing reflex, extubation was performed and the sheep were moved to the postoperative recovery box for 1 day. Postoperatively the sheep were continuously observed until complete recovery of all reflexes. They were administered carprofen (i.m., Rimadyl^®^, Zoetis Deutschland GmbH, Berlin, Germany) in a dose of 4 mg/kg BW daily for 3–5 days to prevent postoperative pain and all animals were examined daily.

Graft patency was monitored immediately after implantation and thereafter at weekly intervals using a bidirectional Doppler measurement (Handydop, ELCAT GmbH, Wolfertshausen, Germany) or color-coded duplex ultrasonography (Vivid i BT10, GE healthcare, Solingen, Germany) (Fig. 6a).

### Explantation

In each treatment group, half of the animals were euthanized at 2 weeks and the other half at 8 weeks after implantation. The sheep were pre-medicated with a pre-mixed solution of 0.16 mg/kg atropine (ATROPINSULFAT 100 mg) and 0.2 mg/kg xylazine (XYLAZIN 2%) and anaesthetized with 2.6 mg/kg propofol (Propofol 1% MCT Fresenius). They were intubated, and anaesthetized with Isoflurane 1.5 vol% and 0.02 mg/kg/h fentanyl (i.v., FENTANYL). Grafts and the adjacent vessels were exposed. The blood flow in the carotid artery was measured using a Transonic^®^ Animal Research Flowmeter T 206 (Transonic Systems Inc., Ithaca, NY, USA). The perivascular ultrasonic volume flow-sensor (6S perivascular probe) was placed around the carotid artery cranially and caudally to the prosthesis (Fig. 5b). The flow was detected by means of the Doppler effect and the average blood flow was displayed on the monitor. Thereafter, the sheep were euthanized with an overdose of sodium pentobarbital (80–90 mg/kg BW, i.v., Narcoren^®^, Boehringer Ingelheim Vetmedica GmbH, Ingelheim, Germany). The carotid artery segments, and -in case of arteriovenous prosthesis shunts- the jugular vein segments were ligated well beyond the anastomoses.

### Post mortem examination

Post mortem examination with special reference to lungs, heart, liver, kidneys, spleen, intestine and brain was done and specimens of these organs and tissues with lesions were taken and immediately fixed in 10% neutral buffered formalin and were processed for histopathological examination as described below.

### Pathological, histopathological and immunohistochemical analyses

For histopathological assessment, the prosthesis materials were sampled together with the adjacent artery or vein and the cranial side was marked. The grafts were macroscopically examined for signs of infection, patency, aneurysm formation and engraftment and were rinsed gently with saline solution. Cross sections were made in three regions of the explanted grafts: caudal, middle, cranial area of the graft.

These specimens were also immediately fixed in 10% neutral buffered formalin, washed in tap water, dehydrated in alcohol, cleared in xylene, embedded in paraffin, sectioned in 2–4 μm slices using a microtome and stained for microscopic examination. Three regions of the patch-angioplasty, interposition graft or shunt (caudal, middle, cranial) were evaluated.

The following parameters were examined: Thrombogenicity, endothelialization, proliferation, biocompatibility, inflammatory cell infiltration and thickness of neointima using hematoxylin and eosin (H&E), Elastica van Gieson (EvG), CD31 and Ki67 staining techniques. A Leica DM 2500 microscope (Leica, Wetzlar, Germany) was used to assess the following parameters: Endothelial cell proliferation, hemorrhage, inflammation, congestion, edema, neutrophils, macrophages and other mononuclear cells, giant cells, myofibroblasts, fibrous connective tissue, capillary proliferation, infection, necrosis, degenerative changes, calcification, thrombus formation.

The microscopic changes to normal physiological findings were evaluated in all stained tissues for the above listed parameters and were graded on a scale from 1 to 4:

1: no changes or negligible

2: mild changes

3: moderate changes

4: severe changes

The scoring was carried out for patch angioplasty in five high power fields (HPF) (2 HPF at junctions (right/left), 3 HPF in the middle of the patches) and for interposition grafts and straight shunts in a minimum of 5 HPF. All evaluations were performed at a magnification of 400. Average score was calculated.

*Elastica van Gieson staining (EvG)* For histochemical staining of elastic fibers, a standard Elastica van Gieson staining protocol was used. Elastic fibers appear red-yellow, while collagen is stained red, and other tissue elements are brownish. In EVG stained samples immature and mature fibroblasts can be differentiated and scored at the inner, middle and outer site of the patch or graft material. 3 HPF within patch materials and 4 HPF within graft interpositions and shunts were evaluated. The presence of immature and mature fibroblasts in the patch or graft material was scored on a scale from 1 to 4:

1: absent or negligible

2: mild

3: moderate

4: high presence

Average score was calculated.

*Immunohistochemistry (CD31, Ki67)* Endothelial cells and proliferating cells were identified by immunohistochemical staining of paraffin-imbedded tissue sections for CD31 and Ki67 expression. Tissue sections were subjected to antigen retrieval by heating the sections for 8 min using a microwave (600 W) in 0.1 mol/L of citrate buffer (pH 6.0) followed by a slow cool down cycle to room temperature. Nonspecific binding sites were blocked based on the primary antibody used with 5% normal goat or rabbit serum in a 2% skim milk solution in phosphate buffered saline (PBS) for 60 min. The sections were incubated overnight at 4 °C with a polyclonal rabbit anti-human CD31 antibody (1:500; antikörper-online.de; ABIN1582260) or a monoclonal mouse anti-human Ki67 MIB 1 antibody (1:75; DAKO, Hamburg, Germany; M7240) at 4 °C. Afterwards sections were incubated with a 1:300 dilution of a biotin-labeled goat anti-rabbit (DAKO; E0432) or rabbit anti-mouse secondary antibody (DAKO; E0413) for 30 min at room temperature. Finally, the slides were incubated with the DAB substrate (Sigma Aldrich, D5905) for 5 min before undergoing Mayer’s Hematoxylin counterstaining for 60 s and being mounted.

Endothelialization was evaluated in CD31 stained tissue slides in 3 HPF at the luminal site of the patch and in 4 HPF of the luminal site of the graft interposition or shunt at a magnification of 400 on a scale from 1 to 4:

1: complete/nearly complete endothelialization (complete lining of CD31 + cells)

2: almost full endothelialization (incomplete CD31 + cell lining)

3: partial endothelialization (some CD31 + cells)

4: no endothelialization (absence of CD31 + cells)

Average score was calculated.

Proliferation of cells was measured in the patch, graft interposition and shunt material by evaluating the number of Ki67 positive cells at a magnification of 400. 5 HPF were counted and average score and standard deviations (SD) were calculated.

All tissue sections were examined in a blinded fashion by an independent board certified pathologist (MA).

## Results

### Health monitoring and acclimatization

**Fig. 4:**
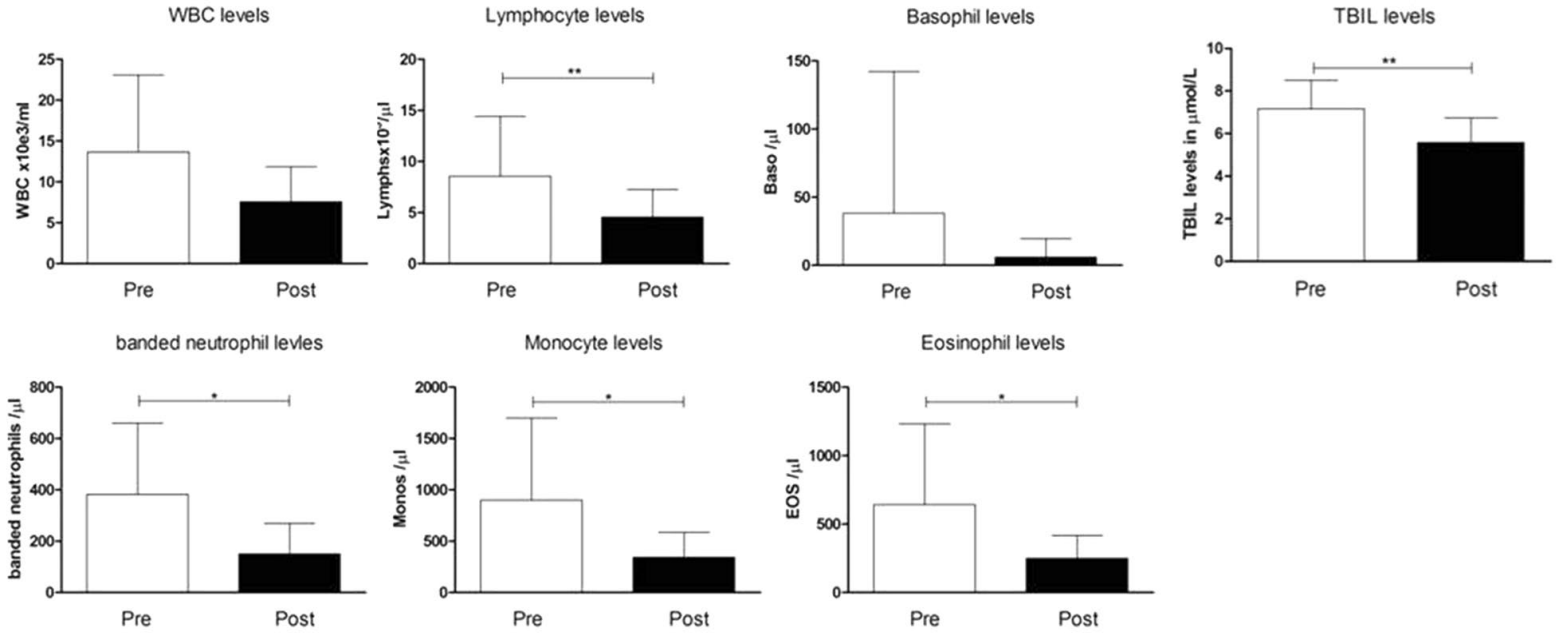
Levels of white blood cells, lymphocytes, basophils, banded neutrophils, monocytes, eosinophils and total bilirubin (TBIL) before (Pre) and after (Post) treatment with albendazol

Although all 24 animals were in good clinical condition at the initial examination, analysis of the fecal samples revealed parasitic infestation of some animals. The following endoparasites were detected in feces: *Ostertagia* (4 animals), T*richostrongylus* (3), *Paramphistomum cervi* (6), *Emeria* spp. (1), *Fasciola hepatica* (3), *Chabertia ovina* (1). *F. hepatica* infestations were detected also in the bile duct at necropsy causing fibrotic cholangitis with calcifications in the liver. In addition, in all 24 animals *Sarcosporidia* were detected in histopathological specimen of the heart. All animals tested negative for serum antibodies against *Corynebacterium pseudotuberculosis*. In accordance with these findings the levels of white blood cells, lymphocytes, banded neutrophils, monocytes, eosinophils and basophils as well as the total bilirubin (TBIL) level in serum were elevated at the time point of arrival (Pre). After treatment with albendazole (Valbazen^®^ 1.9%) within the acclimatization time of 4–8 weeks (Post) these levels went back to base line levels (Fig. 4).

### Implantation

Patches, interposition grafts and straight shunts were implanted bilaterally in the 24 animals with an average operation time of 136 ± 21 min.

*Intra*- *and postoperative complications* All grafts were successfully implanted without any complications during the implantation period. Regurgitation or aspiration during the operation did not occur and the sheep were able to stand or move approximately 15 min after implantation.

During the observed time period for up to 8 weeks the sheep appeared in good health with stable BW (59 ± 6 kg). Two postoperative problems associated with wound healing occurred: wound dehiscence and serous secretion. These wounds were rinsed with betaisodona antiseptic liquid solution (povidone-iodine solution, Mundipharma GmbH, Limburg, Germany) and healed by granulation. Seven sheep developed postoperative local edema and slight hematoma. None of the animals developed clinically apparent infections. The shunts caused no apparent adverse effects on the cardio-circulatory system, as for example pressure decrease, cardiac dilation or cardiac hypertrophy.

*Survival* One sheep with interposition grafts scheduled for an observational period of 2 weeks suffered from ataxia due to bilateral cerebral thromboembolic complications and was sacrificed on the 9th postoperative day. Therefore, the survival in the scheduled observation period was 23/24.

**Fig. 5:**
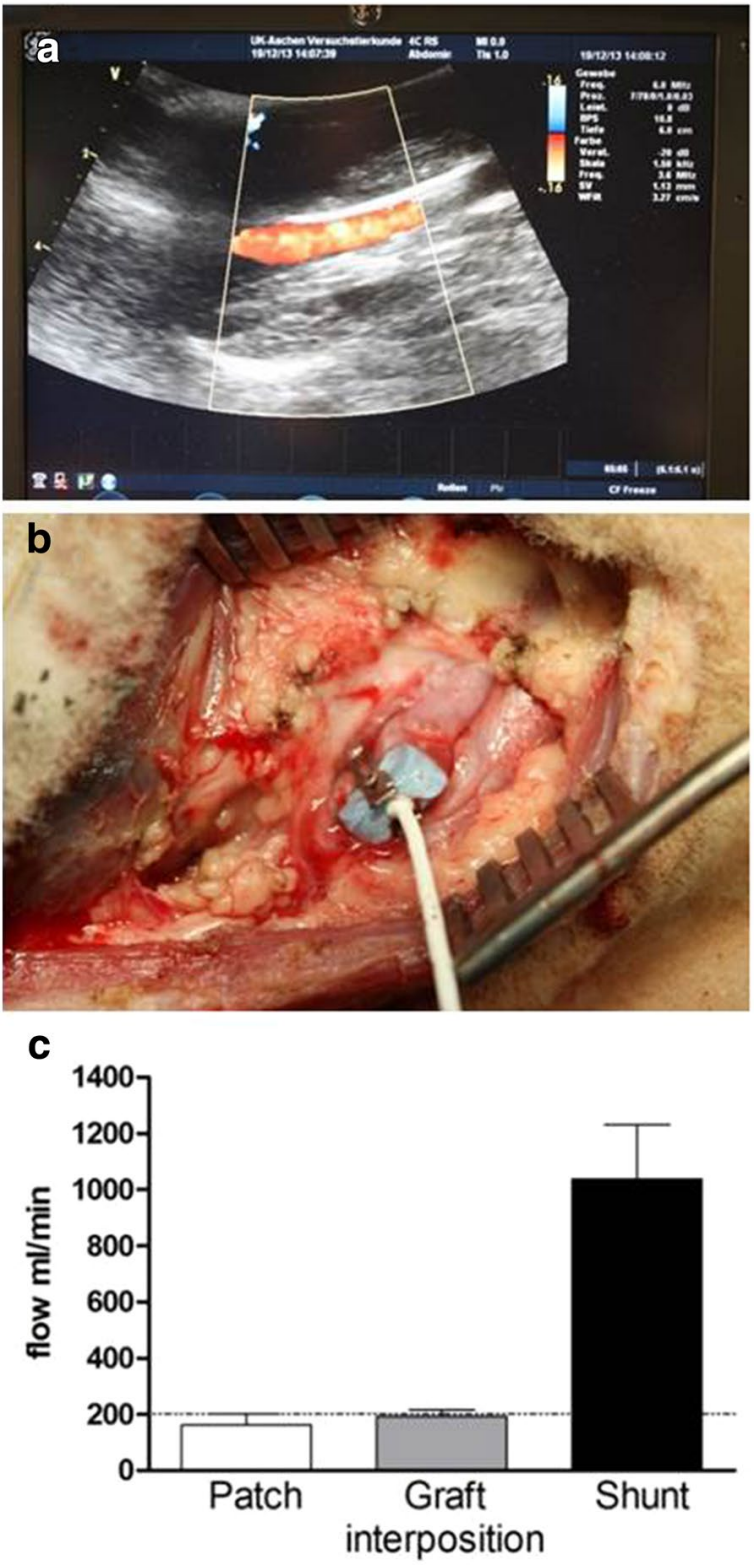
**a** Color-coded duplex ultrasonography of the common carotid artery. **b** Blood flow measurement in the carotid artery. The perivascular ultrasonic volume flow-sensor is placed around the carotid artery. **c** Blood flow at the time point of euthanasia in the common carotid artery after patch angioplasty, graft interposition and straight shunt between the common carotid artery and the external jugular vein. Dotted line represents physiologic blood flow as described in literature

*Graft patency* Two interposition grafts occluded in the above mentioned sheep before the scheduled sacrifice time. All other grafts were patent as the weekly color-coded duplex ultrasonography (Fig. 5a) and bidirectional handheld Doppler measurement (data not shown) demonstrated.

### Explantation

*Blood flow at the time point of euthanasia* After patch angioplasty the blood flow in the cranial part of the common carotid artery was 165.2 ± 41.9 mL/min and in the caudal part was 158.3 ± 40.9 mL/min, with no statistically significant difference between these positions. After graft interposition, the blood flow in the same anatomical regions was 193.2 ± 29.8 mL/min and 190.8 ± 20.2 mL/min respectively, also without any significant difference between both positions (Fig. 5b, c). These data are in accordance to the measurements of physiologic blood flow (202 ± 32 mL/min) in the common carotid artery performed by Baldwin et al. [30]. The volume flow in the straight shunt was 1037 ± 193 mL/min; this value corresponds to the normal value of prosthesis shunts in clinical situations [31].

**Fig. 6:**
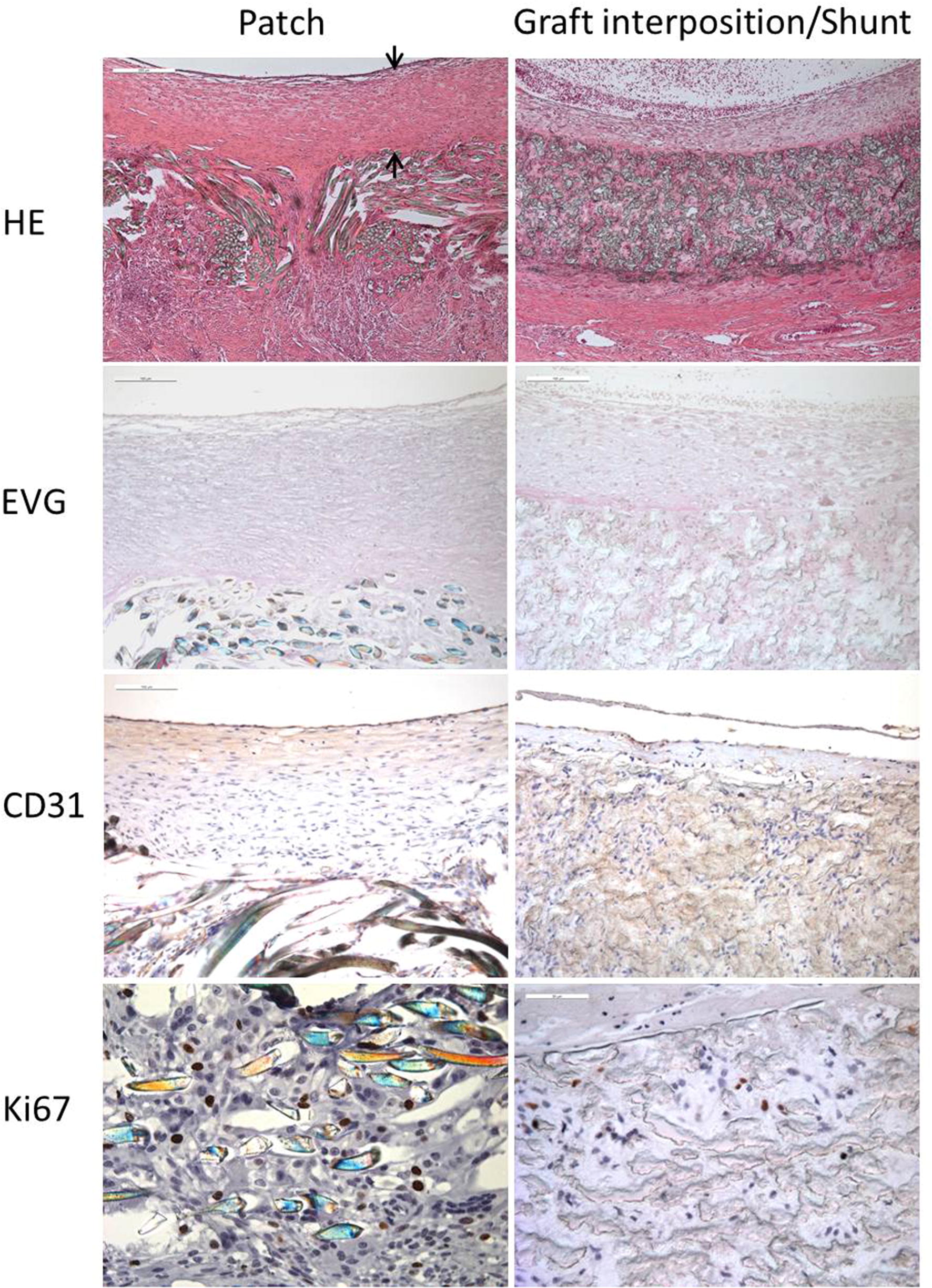
Histology (HE, EVG staining) and immunohistochemistry (CD31, Ki67) after carotid patch angioplasty and graft interposition or shunt between the common carotid artery and the external jugular vein

Histopathological and immunohistochemical (IHC) results are illustrated in Fig. 6. Morphology and structure of the patch and graft material including cell infiltration of various cells could be clearly shown in H&E stainings.

In EvG stained tissues myofibroblast proliferation, maturation and the manner of elastic fiber distribution could be verified.

IHC staining of CD31 positive cells demonstrated the presence of endothelial cells measuring the degree of endothelialization of the graft materials and the level of neo-angiogenesis.

Proliferation of myofibroblasts, endothelial and inflammatory cells measured by Ki67 expression could be demonstrated mainly within the graft material. No proliferation of inflammatory or endothelial cells was observed around the grafts and the surrounding tissue or in the neointima at the time points of 2 and 8 weeks after implantation.

## Discussion

Preclinical animal studies are a prerequisite to assess biocompatibility, thrombogenicity, endothelialization as well as physiologic function of new vascular prosthesis materials prior to clinical trials. In this article we describe our pre- and postoperative care, management of anesthesia and operation procedure step by step and explain the handling of carotid operations in sheep. We performed carotid patch-angioplasties, graft interpositions and arteriovenous prosthetic shunts between the common carotid artery and the external jugular vein without any intraoperative complication and with good mid-term results.

Sheep are considered as a suitable model for vascular surgery because of their comparable vessels size to humans as well as the ease of handling. Their vascular anatomy and biological response to mechanical vessel injury or other interventions resemble those of humans. Sheep develop lesions of intimal hyperplasia that are very similar to those found in failed human access grafts [23, 32–34]. Furthermore, the ovine coagulation system resembles human coagulation better than either dogs or pigs [23, 34, 35]. In general, sheep can be handled more easily for applications and follow-up examinations with ultrasound and Doppler than pigs, which have to be trained prior the experiments or sedated for these procedures, which can induce stress. Pigs respond to stress with hypertension associated with a higher risk of bleeding and subsequently peri-prosthetic hematomas. Hematomas can cause wound infections and impair engraftment. Altogether, sheep therefore are the large animal of choice for the assessment of vascular prostheses.

However, sheep display a tendency to hypercoagulability. Therefore, a high dose of anticoagulant medication must be administered during surgery and the sheep can be anti-coagulated successfully with heparin [34, 36, 37]. As a consequence, we administered 5000 IU of heparin before graft implantation. Before the implantation on the opposite side the injection of heparin was repeated, but with a dose of 2500 IU. With this concept, we achieved a good patency rate. It is known, that in sheep the response to clopidogrel (e.g. Plavix^®^) is modest, and acetylsalicylic acid (e.g. Aspirin^®^) fails to inhibit platelet aggregation [36, 38]. Therefore, we did not administer platelet aggregation inhibitors, which are usually given after clinical carotid surgery.

The location of the graft in the sheep’s neck allows the use of grafts with the same size as used in patients. Sheep carotid arteries are readily accessible and similar in diameter to human peripheral arteries. These criteria also apply to the sheep’s femoral artery. However, operations in the groin are associated with a higher risk of infection and animals are better able to manipulate the surgical wound. In contrast, implantation of grafts in the neck is well tolerated, with minimal postoperative morbidity. Neurologic complications resulting from either clamping during graft implantation or postoperative occlusion are rare due to vertebral arteries, which communicate with the distal common carotid artery through the occipito-vertebral anastomosis and due to the rete mirabilis, a dense network of blood vessels, which is situated high up in the middle of the cranium of the sheep [30, 39–42]. Occlusion of one or both common carotid arteries causes a compensatory increase in blood flow in the contralateral common carotid artery and/or vertebral arteries [30, 42]. To avoid any intraoperative neurologic complications, we performed the graft implantations sequentially, first in one carotid artery, afterwards in the contralateral carotid artery. With the above-described concept of anticoagulation, we achieved good patency rates. In our experiments, only one sheep suffered from ataxia due to bilateral thrombotic interposition graft occlusion and cerebral embolism.

In the literature, observation periods of 2 h [7]—6 m [5] are reported to examine prosthetic graft material after implantation. In accordance with Kohler et al. [23], who performed arteriovenous prosthetic shunts and observed the development of intimal hyperplasia, especially at the venous anastomoses within 2 and 8 weeks, we also chose an observation period of 2 and 8 weeks.

Kohler et al. [23] performed arteriovenous cross over shunts between the common carotid artery and the contralateral external jugular vein. They observed the development of neointimal hyperplasia, especially at the venous anastomosis. However, in order to examine new prosthesis material, we performed arteriovenous straight shunts between the common carotid artery and the ipsilateral external jugular vein. In this manner, we could implant two different prosthesis materials on both sides. Implantation of the control prosthesis in the same sheep reduces the effect of inter-individual variability and allows paired significance tests, minimizing the number of animals required.

In our experiments, the shunts were not punctured. Therefore, we were able to guide the straight prosthesis shunt under the sternocleidomastoid muscle. In this manner less turbulence occurred. However, if a shunt puncture is planned, the prosthesis must be guided in loop form over the sternocleidomastoid muscle.

Of course, the results of animal experiments are not fully translatable to humans. For example, the quality and velocity of tissue integration or development of neointimal hyperplasia cannot be transferred completely. Furthermore, in our study, healthy vessels were operated instead of vessels with arteriosclerotic plaques. Therefore, before patch-angioplasty, no thrombendarteriectomy was performed as usual in humans.

The here described successive evaluation of graft material which was tested first as a patch and then translated into graft interposition and finally evaluated as a shunt addresses the 3R principle by minimizing the risk of serious side effects, e.g. accompanied with thrombus formation due to the graft material tested.

## Conclusion

Transfer of the results concerning vascular prosthesis materials from sheep to humans requires additional clinical studies before its use in clinical practice.

Here, we describe step by step our pre- and postoperative care, management of anesthesia and operation procedures. Furthermore, we explain tips and tricks for handling carotid artery and jugular vein operations in sheep to avoid possible pitfalls, frustrations and loss of animals. Following our comprehensive recommendations a remarkable reduction and refinement of animal use can be realized. Furthermore, standardized protocols may improve reproducibility and transparency, when different research groups perform experiments.

## Abbreviations

ALB: albumin
ALP: alkaline phosphatase
ALT: alanine aminotransferase
AMYL: amylase
AST: aspartate aminotransferase
BUN: blood urea nitrogen
Ca: calcium
CHOL: cholesterol
CK: creatine kinase
Cl: chloride
CREA: creatinine
CRP: C-reactive protein
GGT: gamma glutamyl transferase
GLU: glucose
h: hours
HPF: hight power field
i.m.: intramuscular
i.v.: intravenous
K: potassium
LAC: lactose
LDH: lactate dehydrogenase
LIP: lipase
m: month
Mg: magnesium
min: minute
mL: milliliter
Na: sodium
PHOS: phosphorus
TBIL: total bilirubin
TP: total protein
TRIG: triglycerides
UAC: uric acid
